# Design and performance analysis of a simplified hybrid modulation method for dual active bridge powering inverters

**DOI:** 10.1371/journal.pone.0341443

**Published:** 2026-02-03

**Authors:** Mohamad Fathi Mohamad Elias, Intan Mastura Saadon, Norridah Amin

**Affiliations:** 1 Higher Institution Centre of Excellence (HICoE), UM Power Energy Dedicated Advanced Centre (UMPEDAC), Level 4, Wisma R&D Universiti Malaya, Kuala Lumpur, Malaysia; 2 Faculty of Electrical Technology and Engineering, Universiti Teknikal Malaysia Melaka, Durian Tunggal, Melaka, Malaysia; PLOS, UNITED KINGDOM OF GREAT BRITAIN AND NORTHERN IRELAND

## Abstract

This paper presents a simplified hybrid modulation method for operating dual-active-bridge (DAB) converters that power inverters by integrating single-phase shift (SPS) and triple-phase shift (TPS) modulation schemes. It covers the design and control algorithm development, performance analysis, as well as highlights its benefits and limitations. While full TPS implementation is highly complex, this work selects a specific TPS operating mode to enhance DAB converter efficiency in low-power conditions with minimal control effort. In the proposed method, a hysteresis controller is employed to regulate the DAB modulation at a defined power threshold. This poses a significant challenge, especially when a single proportional-integral (PI) controller is employed to regulate output power with a minimal set of control parameters applicable to both modulation schemes. Moreover, in addressing these challenges, a trade-off between high efficiency and fast dynamic response is also considered, with greater emphasis placed on efficiency when developing the controllers. Meanwhile, the inverter output voltage is independently controlled regardless of the DAB operation to further simplify the overall process. Small signal modeling and closed-loop control of DAB-based inverter with the proposed hybrid modulation are also presented. Its functionality and performance have been verified through simulation and a developed small-scale DAB-based inverter prototype.

## 1 Introduction

For a sustainable future, it is important to maximize the utilization of clean and renewable energy sources in all sectors while reducing reliance on fossil fuels. Wind and solar energy are among the best options owing to easier implementation and lower cost. In small to large-scale grid-connected applications, both sources typically require no storage devices because all harvested energy is exported directly to the grid. Meanwhile, in stand-alone applications where the load consumption and renewable power generation are generally unmatched, energy storage devices such as secondary batteries are installed to store excess energy generation and release energy when needed. Focusing on the small to medium-scale standalone solar photovoltaics (PV) applications, the generated energy is typically stored in a battery pack using a charge controller, and to produce a standard AC voltage, the battery voltage is increased to a relatively higher DC voltage by using a DC-DC converter before being converted into AC voltage by using an inverter.

For this purpose, a dual active bridge (DAB) converter is an excellent choice due to its high energy conversion efficiency, high power density, bidirectional power transfer capability, and galvanic isolation. These advantages make the DAB converters attractive and suitable for various applications such as solid-state transformers [1,2], electric vehicle chargers [3], uninterrupted power supplies (UPS) [4], and DC microgrids [5]. Modulation methods applicable for DAB converters are single-phase shift (SPS) [6], extended phase shift (EPS) [7], dual-phase shift (DPS) [8], and triple-phase shift (TPS) [9–12] modulations. From the literature, all these modulations are operated independently for various applications. In comparison, SPS is the most commonly used and simplest among all methods because it requires only one parameter to control, which is the phase shift. In a closed-loop operation, a controller is required to regulate the output power according to the load demand. Among the controllers that have been proposed for SPS modulation are proportional-integral (PI) [13,14], sliding-mode control (SMC) [15,16], and model predictive control (MPC) [17,18]. On the other hand, TPS is the most advanced method that offers higher efficiency and flexibility, but at the expense of higher complexity, especially in real-time implementation with another 2 control parameters in addition to phase shift for controlling the converter. For optimal operation, a different power level has a unique set of parameters, which is typically acquired through a lookup table or formula calculation [13]. In [19], TPS modulation has been used together with a PI controller to control the output power and to regulate the DC output voltage. The PI controller generates the reference power that will be compared with the threshold power to determine between two TPS operating modes, low-power or high-power mode. In this case, further computations are still needed to calculate the three parameters after the controller generates control output, and hence delaying the whole process.

An efficiency-oriented automatic triple phase shift (ATPS) modulation has been proposed in [20], which involved 3 stages. In Stage 1, simulation is performed in PLECS to obtain power loss performance based on the output power, output voltage, and duty cycles of primary and secondary full bridges, which is then modeled into a neural network (NN)-based power loss model. Subsequently, the particle swarm optimization (PSO) method is used to optimize the modulation parameters, which are the duty cycles with minimal power loss for the selected combinations of output power and voltage in Stage 2. Stage 3 is the real-time implementation of optimal TPS using the Fuzzy Inference System (FIS) for generating the required duty cycles and the PI controller for generating the phase shift. This method is very dependent on the model of converters for simulation, NN modeling, optimization, and implementation. In [21], the analytical and normalized equations approach is used to implement the optimized TPS modulation strategy. This method employs voltage control in the outer loop to regulate the DC link voltage while power control is in the inner loop. The power control loop requires voltage ratio and minimum zero-voltage-switching current information in addition to a proportional (P) controller to determine the case and the modulation indices for generating the optimum switching signals. Unlike the previous developments that solely implemented TPS throughout the power range, a hybrid modulation combining SPS with the primary-side internal phase shift (PS-IPS) and secondary-side internal phase shift (SS-IPS) is proposed in [22]. The PS-IPS and SS-IPS modulations are used for light load and medium load respectively, whereas SPS is used for heavy load, defined as more than 50% of the rated power. There are two power thresholds to select between PS-IPS and SS-IPS operations, which are determined based on the transfer voltage ratio, while the output power regulation is performed by using a single PI controller for all modulation modes.

In this paper, the DAB operational complexity with full TPS modulation is further reduced with the proposed hybrid modulation method combining a specific TPS modulation mode at low power and SPS modulation for high-power conditions. This method improves converter efficiency at light loads while ensuring straightforward operation at heavy loads, making it well-suited for real-time implementation. A single PI controller is used for output power regulation, while a hysteresis controller is used to control the transition between TPS and SPS modes. To the best of the authors’ knowledge, no investigation on the DAB operation using the proposed hybrid modulation method has been presented in the literature. Moreover, the focus is mostly on DAB operations with their proposed modulations under the same ratio, low step-up or step-down operations. Meanwhile, in this paper, the focus is on high step-up DAB operations with an inverter load. The effect of second-order harmonics on the DAB voltage regulation has been considered, including a compromise between high efficiency and fast dynamic response of the whole system, in which the emphasis is more on achieving higher efficiency with reduced complexity. This paper is organized as follows. Section 2 presents the proposed simplified hybrid modulation method, including small signal modeling of the DAB with inverter load. Simulations of the proposed hybrid modulation are presented in Section 3. Section 4 explains the DAB-based inverter prototype development, including the design specifications. Section 5 presents the experimental results showing the voltage and current waveforms of the DAB operating under TPS and SPS modulations and inverter outputs, the DC to AC conversion efficiency at various power levels, as well as the transient performance during step changes in load. Finally, the conclusion is given in Section 6.

## 2 Proposed simplified hybrid modulation method for DAB converters

**Fig 1:**
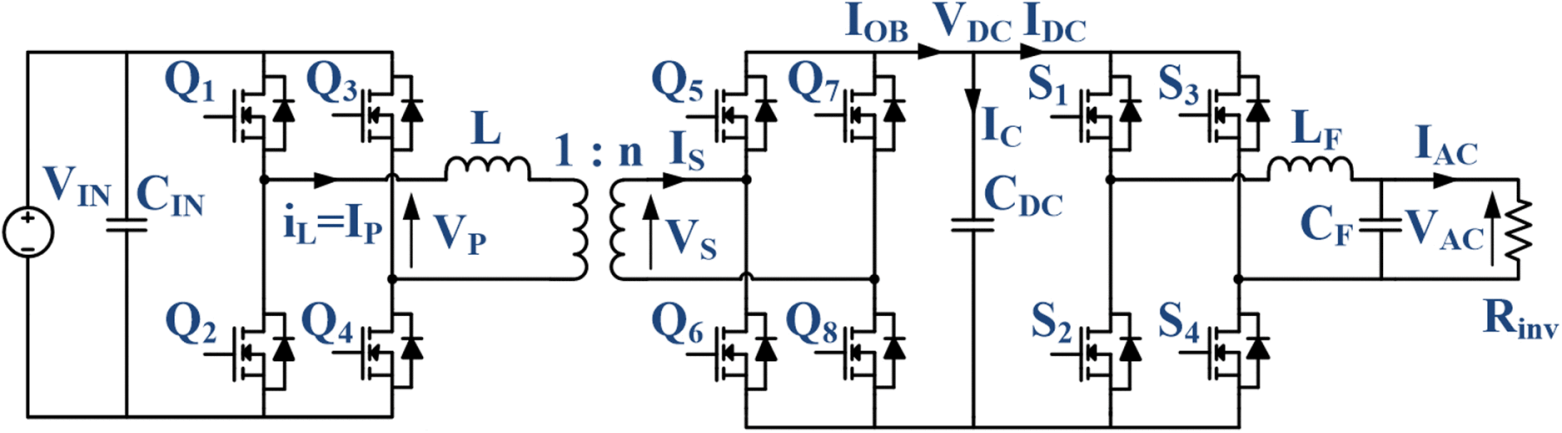
Schematic circuit of DAB-based inverter.

The circuit topology showing the DAB driving a single-phase inverter is shown in Fig 1. This topology is chosen since the focus is on a standalone solar PV application. In a standalone PV system, battery storage is required to buffer the intermittency in solar power generation. Accordingly, the converter will only see a constant DC power supply, and hence the PV module input is excluded from the analysis. The inverter load is included since it affects the DAB output voltage regulation by creating second-order harmonics.

### 2.1 Design of simplified hybrid modulation method

The simplified hybrid modulation method aims to improve the efficiency of the DAB converter at low power conditions by employing TPS modulation while maintaining simple control at high power conditions using SPS modulation. From the literature, there are a few modulation schemes applicable for DAB converters, namely single-phase shift (SPS), extended phase shift (EPS), dual-phase shift (DPS), and triple-phase shift (TPS) modulations.

**Fig 2:**
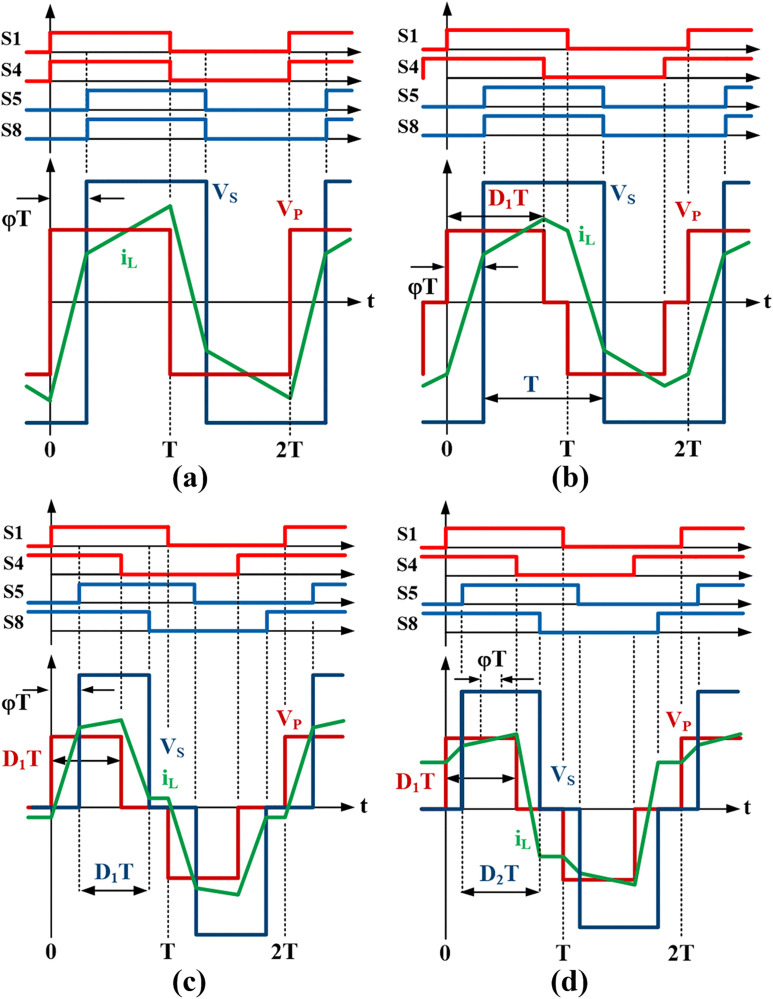
DAB modulation schemes. (a) SPS, (b) EPS, (c) DPS, (d) TPS.

Fig 2 shows the differences between all the modulation methods in terms of the switching signals and the corresponding full-bridge voltage and current waveforms. $S_{\text{1}}-S_{\text{4}}$ are the switching signals for the primary full-bridge, $S_{\text{5}}-S_{\text{8}}$ are the signals for the secondary full-bridge, $V_{P}$ is the primary full-bridge voltage, $V_{S}$ is the secondary full-bridge voltage and $i_{L}$ is the inductor current. From the figure, $D_{\text{1}}$ and $D_{\text{2}}$ represent the duty cycles for the first and the second full bridge respectively, and φ represent the phase shift between the two full bridges. In SPS modulation, $D_{\text{1}}=D_{\text{2}}=\text{1}$, and φ is the only parameter to control the output power. The EPS modulation adds another parameter to control in addition to φ by setting either $D_{\text{1}}$ or $D_{\text{2}}<\text{1}$. In DPS, in addition to φ, duty cycles are set to $D_{\text{1}}=D_{\text{2}}<\text{1}$. Whereas, in TPS modulation, all three parameters can be controlled independently.

The detailed analyses covering all possible switching modes, zero-voltage switching (ZVS) and zero-current switching (ZCS) operations that help to minimize switching power losses for all modulation schemes have been comprehensively discussed in the literature [6–12]. Operation modes with reduced reactive power consumption that minimize the inductor current and lower the current stress of the power switches have also been analyzed. Hence, this paper will focus only on the relevant details of the SPS and TPS modulation to understand the proposed modulation method.

For SPS modulation, the simple average model of DAB is used for analysis [6]. Based on this model, the output average current of DAB can be represented by Eq (1). Hence, by taking the product of output average current and output voltage, the expression of output power can be obtained as Eq (2).

**Fig 3:**
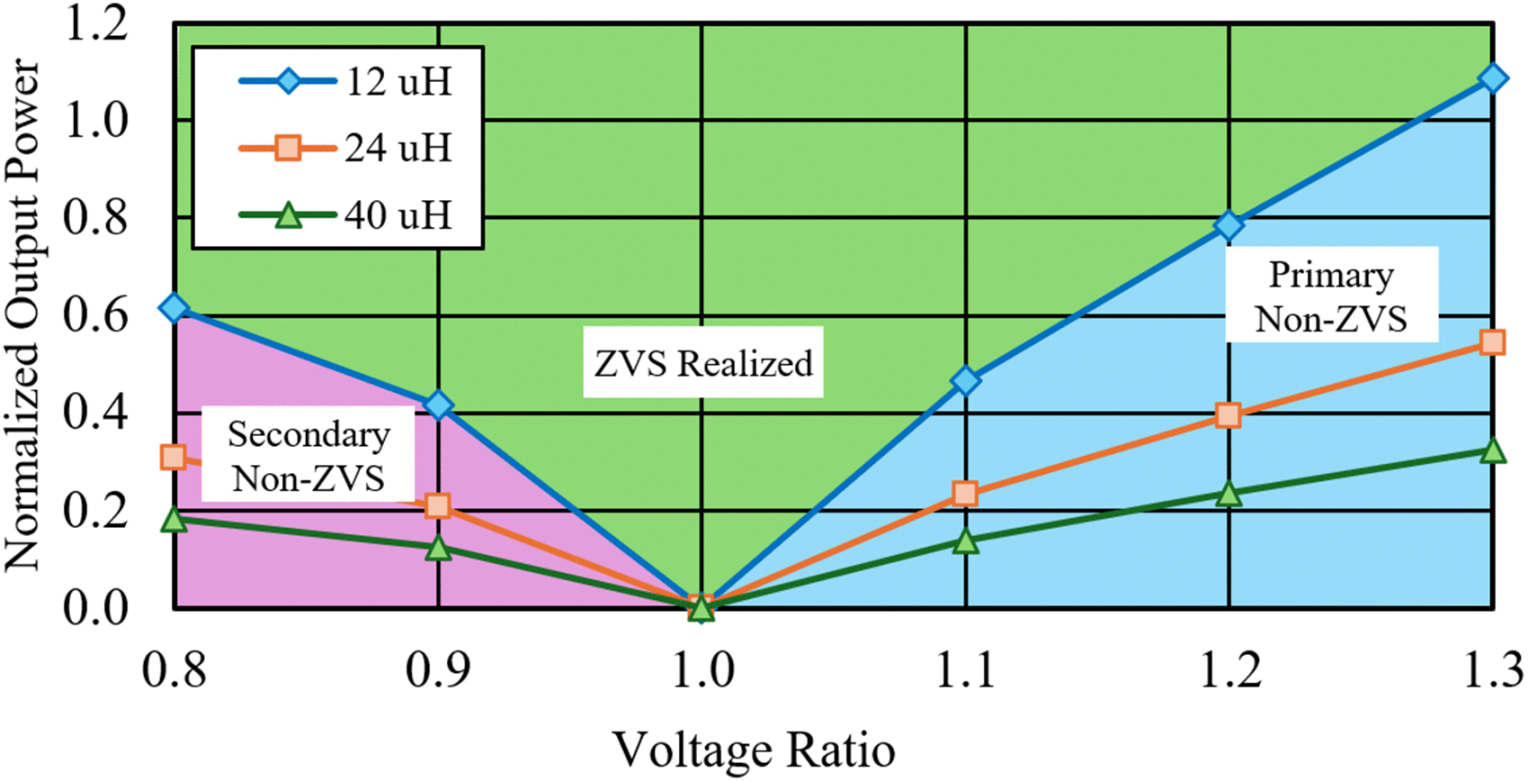
ZVS range and normalized output power versus voltage ratio for SPS.


From Eq (2), the phase shift parameter, φ contributes to the inverse parabolic effect on the output power when it varies from 0 to 1, where the maximum output power is obtained when φ=0.5. To maintain a linearity of control, φ is set to a maximum of 0.35 at the rated power operation. One of the advantages when using DAB is the ZVS operation or soft switching capability that helps in reducing power losses. Typically, the ZVS operation is maintained at medium to high power operations, but decreases towards low power regions. The soft switching operation is also determined by the ratio between the primary and the secondary output voltage, *M* as defined in Eq (3), where *n* is the transformer step-up ratio. Fig 3 shows the ZVS operation region at various power levels and voltage ratios for leakage inductance, L = 12 μH. By maintaining *M* = 1, ZVS can be achieved throughout the power range. From the figure, it can be seen that the ZVS region becomes wider when the leakage inductance value is increased to 24 μH and 40 μH for instance.

$$\overset{―}{I_{DC}}=\frac{(\text{1}-\varphi )\varphi TV_{IN}V_{DC}}{nL}$$ (1)

$$P_{DC}=V_{DC}\overset{―}{I_{DC}}=\frac{(\text{1}-\varphi )\varphi TV_{IN}V_{DC}}{nL}$$ (2)

$$M=\frac{V_{DC}}{nV_{IN}}$$ (3)

$$i_{L}\left(t_{PF}\right)=\frac{TV_{IN}}{\text{2}L}(\text{2}M\varphi +\text{1}-M)$$ (4)

$$i_{L}\left(t_{SR}\right)=\frac{TV_{IN}}{\text{2}L}(\text{2}\varphi -\text{1}+M)$$ (5)

**Fig 4:**
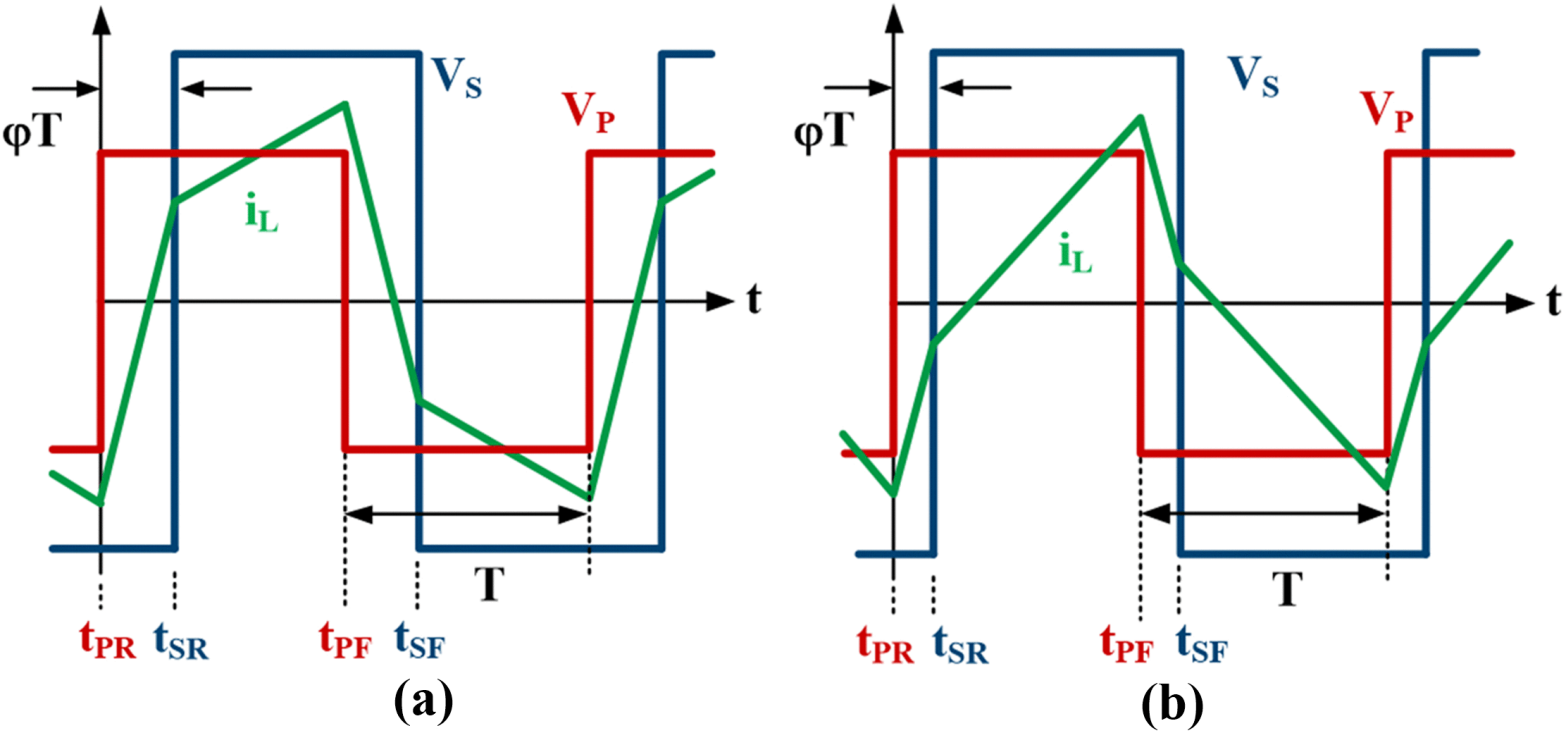
SPS Modulation. (a) ZVS realized (b) ZVS lost during t_SR_ and t_SF_.

To satisfy ZVS operation for both primary and secondary circuits, the inductor current must be negative when the primary voltage, $V_{P}$ is rising and positive when the secondary voltage, $V_{S}$ is rising as shown in Fig 4(a) [8]. As the output power decreases to a very low level, one inductor current condition cannot be fulfilled, in which the inductor current becomes negative when the secondary voltage, $\begin{array}{c} V_{S} \end{array}$ rises as illustrated in Fig 4(b). Hence, this causes the DAB to operate in hard switching and reduce efficiency. The inductor current amplitudes when $V_{P}$ is falling and $\begin{array}{c} V_{S} \end{array}$ is rising can be obtained using Eqs (4) and (5) respectively.

**Fig 5:**
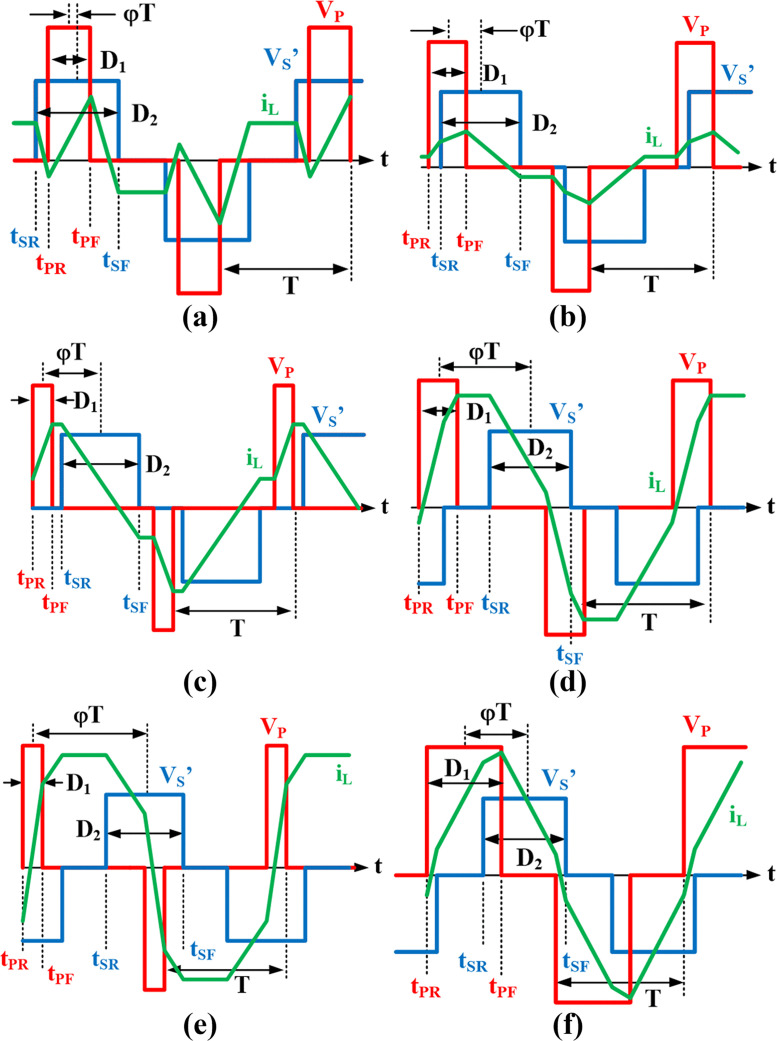
TPS modulation modes for Case II. (a) TPS1 (b) TPS2/TPS2* (c) TPS3 (d) TPS4 (e) TPS5 (f) TPS3*.


To solve this inefficiency issue in low-power conditions, a TPS modulation scheme can be employed. In general, since all the parameters can be varied, the DAB operation can be divided into 4 main cases: Case 1: $V_{P}>V_{S}^{\prime }\;\&\;D_{\text{1}}>D_{\text{2}}$, Case 2: $V_{P}>V_{S}^{\prime }\;\&\;D_{\text{1}}<D_{\text{2}}$, Case 3: $V_{P}<V_{S}^{\prime }\;\&\;D_{\text{1}}>D_{\text{2}}$, and Case 4: $V_{P}<V_{S}^{\prime }\;\&\;D_{\text{1}}<D_{\text{2}}$, with $V_{S}^{\prime }=\frac{V_{S}}{n}$ [23]. In this paper, Case 2 is selected for positive power transfer at low power levels. By varying the phase shift from 0 to 1, the DAB operation can be further divided into 6 operating modes as shown in Fig 5, whereas its boundaries are given in Table 1 [23]. It is noted that there are two variations for operating modes, TPS2 and TPS3. TPS2 and TPS3 are for $D_{\text{1}}<D_{\text{2}}$ and $D_{\text{1}}>\text{1}-D_{\text{2}}$, whereas TPS2* and TPS3* are for $D_{\text{1}}<D_{\text{2}}$ and $D_{\text{1}}<\text{1}-D_{\text{2}}$. Based on the detailed analysis performed in [19], the maximum power swing from TPS3*, followed by TPS4 and TPS5, TPS3, TPS2/TPS2*, and TPS1 for a very low power level. Furthermore, for power less than 25%, TPS2/TPS2* is the optimum mode with reduced core loss, especially in boost mode. The comparative analysis performed in [19] also showed that TPS2/TPS2* operates with the least amount of current for power flow from 0 to 55% of the rated output power as compared to other modes, hence producing higher energy efficiency. Therefore, in this paper, a hybrid scheme combining a specific TPS modulation mode which is TPS2 and SPS will be proposed to simplify the DAB switching operations while maintaining higher efficiency at low power conditions. For modulation mode TPS2 (afterward, will be referred to only as TPS), the boundaries for $D_{\text{1}}$, $D_{\text{2}}$ and φ are defined as Eq (6) [19]. For optimal modulation at low power conditions, the values of $D_{\text{1}}$ and $D_{\text{2}}$ are set as 0.5 and 0.6 respectively [20].

**Table 1:** Boundaries for modulation mode in Case II.

Modulation Mode	Phase Shift Range
TPS1	$\text{0}<\varphi \leq \left(\frac{D_{\text{2}}-D_{\text{1}}}{\text{2}}\right)$
TPS2	$\left(\frac{D_{\text{2}}-D_{\text{1}}}{\text{2}}\right)<\varphi \leq \left(\frac{D_{\text{2}}+D_{\text{1}}}{\text{2}}\right)$
TPS2*	$\left(\frac{D_{\text{2}}-D_{\text{1}}}{\text{2}}\right)<\varphi \leq \left(\text{1}-\frac{D_{\text{2}}+D_{\text{1}}}{\text{2}}\right)$
TPS3	$\left(\frac{D_{\text{2}}+D_{\text{1}}}{\text{2}}\right)<\varphi \leq \left(\text{1}-\frac{D_{\text{2}}+D_{\text{1}}}{\text{2}}\right)$
TPS3*	$\left(\text{1}-\frac{D_{\text{2}}+D_{\text{1}}}{\text{2}}\right)<\varphi \leq \left(\frac{D_{\text{2}}+D_{\text{1}}}{\text{2}}\right)$
TPS4	$\left(\text{1}-\frac{D_{\text{2}}+D_{\text{1}}}{\text{2}}\right)<\varphi \leq \left(\text{1}-\frac{D_{\text{2}}-D_{\text{1}}}{\text{2}}\right)$
TPS5	$\left(\text{1}-\frac{D_{\text{2}}-D_{\text{1}}}{\text{2}}\right)<\varphi \leq \pi$

$$\varphi \leq D_{\text{1}}\leq \pi ,\;\;\text{0}\leq D_{\text{2}}\leq \pi ,\;\;D_{\text{1}}\leq \varphi +D_{\text{2}}\leq \pi$$ (6)

Regarding the soft switching operation of TPS modulation, in general, the ZVS condition is achieved during the turn-on of the power switches $Q_{\text{1}}$, $Q_{\text{4}}$, $Q_{\text{6}}$ and $Q_{\text{7}}$ when $i_{L}>\text{0}$ and in $Q_{\text{2}}$, $Q_{\text{3}}$, $Q_{\text{5}}$ and $Q_{\text{8}}$ when $i_{L}<\text{0}$ by referring to Fig 1. The detailed ZVS conditions for each power switch as a function of inductor current $\begin{array}{c} i_{L} \end{array}$ are given in Table 2. The power equation for the TPS2 mode, which is based on $D_{\text{1}}$, $D_{\text{2}}$ and φ is given by Eq (7) and the corresponding inductor currents are given by Eqs (8) to (10) [23].

**Table 2:** ZVS conditions for each switch.

Switch	ZVS
Q1	$i_{L}\left(t_{PR}\right)<\text{0}$
Q2	$i_{L}\left(t_{PR}+T\right)>\text{0}$
Q3	$i_{L}\left(t_{PF}\right)>\text{0}$
Q4	$i_{L}\left(t_{PF}+T\right)<\text{0}$
Q5	$i_{L}\left(t_{SR}\right)>\text{0}$
Q6	$i_{L}\left(t_{SR}+T\right)<\text{0}$
Q7	$i_{L}\left(t_{SF}\right)<\text{0}$
Q8	$i_{L}\left(t_{SF}+T\right)>\text{0}$

**Fig 6:**
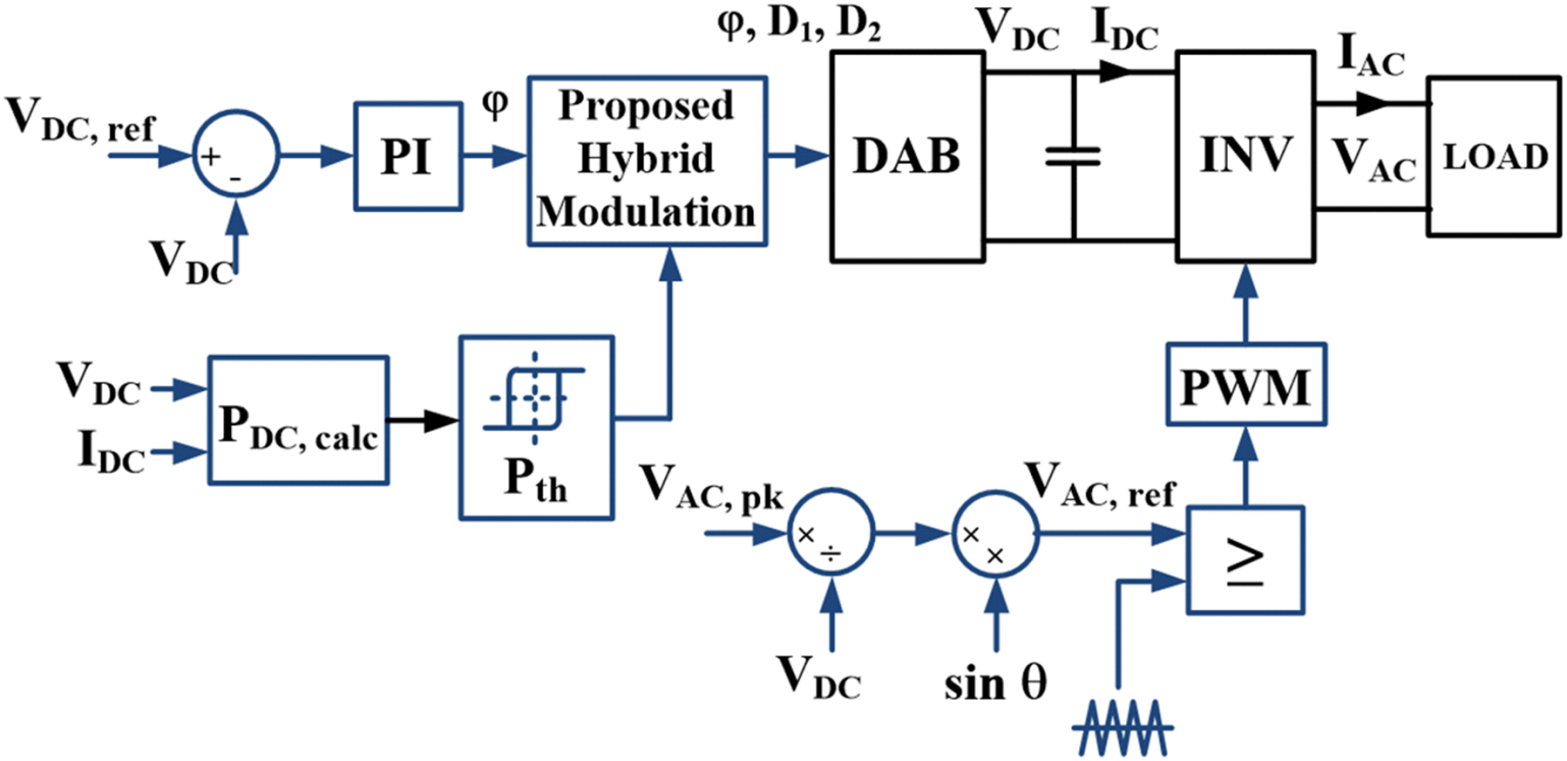
Control block diagram of the proposed simplified hybrid modulation with sinusoidal PWM switching.


Fig 6 shows the control block diagram for the DAB driving a single-phase inverter using the simplified hybrid modulation method. For simplicity, the DAB and the inverter are independently controlled. The DAB is controlled by using the proposed hybrid modulation method to provide a fixed DC link voltage, $\begin{array}{c} V_{DC} \end{array}$ for the inverter. Within the DAB control loop, the DC link voltage and current will be measured to determine the DC output power.

$$P_{DC}=\frac{V_{IN}V_{DC}}{\text{4}LF_{s}n}\left[\varphi \left(D_{\text{1}}+D_{\text{2}}-\varphi \right)-\frac{{\left(D_{\text{1}}-D_{\text{2}}\right)}^{\text{2}}}{\text{4}}\right]$$ (7)

$$i_{L}\left(t_{PR}\right)=-i_{L}\left(t_{SF}\right)=-\frac{D_{\text{1}}V_{IN}n-D_{\text{2}}V_{DC}}{\text{4}LF_{s}n}$$ (8)

$$i_{L}\left(t_{PF}\right)=\frac{D_{\text{1}}V_{IN}n+\text{2}V_{DC}\varphi -D_{\text{1}}V_{DC}}{\text{4}LF_{s}n}$$ (9)

$$i_{L}\left(t_{SR}\right)=-\frac{D_{\text{2}}V_{IN}n-\text{2}V_{IN}\varphi n-D_{\text{2}}V_{DC}}{\text{4}LF_{s}n}$$ (10)

The hysteresis controller is used to determine the optimum modulation mode based on a defined power limit. In this case, TPS and SPS modulations will be used for output power below and above 30% respectively. A power boundary of 30% is selected based on partitioning the DAB operation range into low, medium, and high-power levels, where each represents approximately one-third of the rated output power. The DAB output power is controlled by using the phase shift between the primary and secondary full bridges in both modulation modes. A proportional-integral (PI) controller is used to generate the required phase shift based on the difference between the reference and the actual $V_{DC}$ values. The closed-loop control is also employed for the inverter to regulate the AC output voltage throughout the power range. In the inverter control loop, the peak amplitude of the desired $V_{AC}$ and the measured $V_{DC}$ are used to generate the modulating signal for the inverter. The PWM signals are generated by comparing the modulating reference signal with the triangular carrier based on the unipolar PWM method.

**Fig 7:**
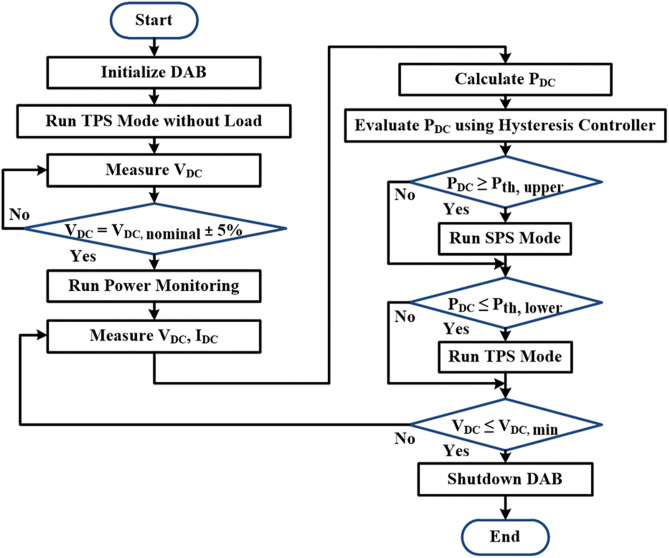
Flowchart of the proposed simplified hybrid modulation method.

The flowchart of the proposed modulation method for the DAB operation is shown in Fig 7. The first step involves initialization by running the DAB under TPS mode without load until the DC link voltage stabilizes within 5% of its nominal value. Upon this condition being achieved, the power monitoring loop will be enabled. In the proposed simplified hybrid modulation method, the DC link voltage and current will always be monitored to obtain the real-time output power and fed to the hysteresis controller for optimum modulation mode determination. The hysteresis controller is used to prevent frequent switching between TPS and SPS modes at the power boundary, which is set to 30% of the rated power. The hysteresis controller limits are defined as ±5% of the established power boundary. Accordingly, the system transitions from TPS to SPS when the output power exceeds the upper limit $P_{th,\;\;upper}$, 31.5% and reverts to TPS when it falls below the lower limit $P_{th,lower}$, 28.5%. Effectively, the hysteresis bandwidth corresponds to only ±1.5% of the rated output power. In the case where the DC link voltage decreases below the minimum DC input voltage required by the inverter, the DAB will be turned off. The main procedures for identifying the DAB operating mode within the power monitoring loop are presented in the pseudocode below.

1: **loop** continuously:

2:  measure DC link voltage

3:  measure DC link current

4:  calculate power

5:  **if** power > upper power limit **then**

6:   set to operate in SPS mode

7:  **else if** power < lower power limit **then**

8:   set to operate in TPS mode

9:   **end if**

10: **if** TPS mode **then**

11:  execute TPS switching control

12: **else if** SPS mode then

13:  execute SPS switching control

14: **end if**

15: **end loop**

### 2.2 Small signal model and closed-loop control

**Fig 8:**
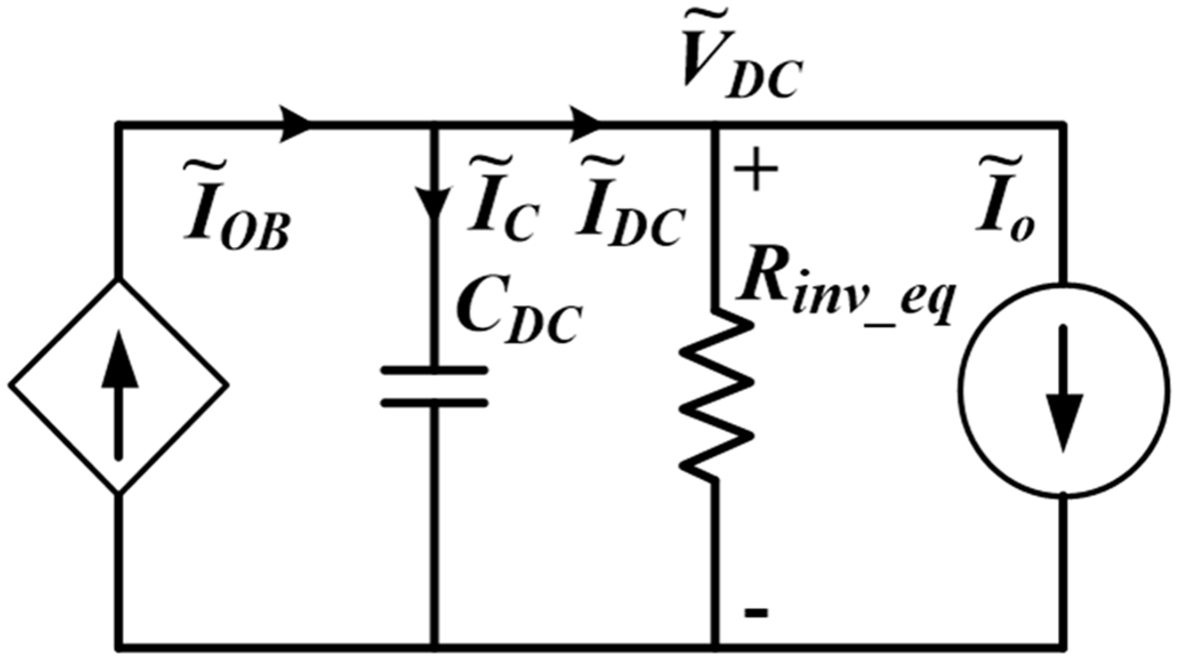
Small-signal model of DAB with an inverter load.


The DAB driving an inverter load can be modeled by using the small-signal average model as shown in Fig 8 [24], where ${\tilde{I}}_{OB}$ is the output bridge current, $C_{DC}$ is the DC link capacitor, $R_{inv\_eq}$ is the inverter load modeled as an equivalent DC resistance, ${\tilde{I}}_{DC}$ is the load current and ${\tilde{I}}_{o}$ is the small signal perturbation comprising second-order harmonics due to the inverter load. The average output current $I_{OB}$ can be derived from the output power expression. Since the DAB operations involve SPS and TPS modulations, which depend on the power levels, two small signal models are used. From Eqs (2) and (7), the $I_{OB}$ for SPS and TPS modulations can be derived as Eqs (11) and (12) respectively. It is noted that the small-signal average model has a limited capability as it neglects the high-frequency dynamics of the converter, such as the switching transients. Therefore, the more detailed characteristics of the DAB-based inverter can be obtained through power electronics simulation software, as presented in the next section.

$$I_{OB\_SPS}=\frac{V_{IN}\varphi (\text{1}-\varphi )}{\text{2}LF_{s}n}$$ (11)

$$I_{OB\_TPS}=\frac{V_{IN}}{\text{4}LF_{s}n}\left[\varphi \left(D_{\text{1}}+D_{\text{2}}-\varphi \right)-\frac{{\left(D_{\text{1}}-D_{\text{2}}\right)}^{\text{2}}}{\text{4}}\right]$$ (12)

Assuming all the power is transferred from the DAB to the inverter, the equivalent DC resistance can be obtained as Eq (13).


$$R_{inv\_eq}=\frac{V_{DC}^{\text{2}}R_{inv}}{V_{AC\_rms}^{\text{2}}}$$ (13)

The open-loop small signal control-to-DC link voltage transfer function can be obtained by analyzing Fig 8 for both modulations. From Fig 8, first, the DC link voltage expressions for SPS and TPS modulations are obtained as Eqs (14) and (15) respectively.


$$V_{DC\_SPS}=\frac{V_{IN}\varphi (\text{1}-\varphi )}{\text{2}LF_{s}n}\left[\frac{R_{inv\_eq}}{\text{1}+sR_{inv\_eq}C_{DC}}\right]$$ (14)

$$V_{DC\_TPS}=\frac{V_{IN}}{\text{4}LF_{s}n}\left[\varphi \left(D_{\text{1}}+D_{\text{2}}-\varphi \right)-\frac{{\left(D_{\text{1}}-D_{\text{2}}\right)}^{\text{2}}}{\text{4}}\mathrm{\backslash rightleft}[\frac{R_{inv\_eq}}{\text{1}+sR_{inv\_eq}C_{DC}}\right]$$ (15)

Then, the small signal variation in $V_{DC}$ with respect to variation in phase shift, φ which also represents the control-to-DC link voltage transfer function for each modulation, is derived by taking a partial derivative of Eqs (14) or (15) with respect to φ. The results are given by Eqs (16) and (17) for SPS and TPS modulations respectively. Similarly, from Fig 8 the perturbation current-to-DC link voltage transfer function can be obtained as Eq (18). The perturbation current, ${\tilde{I}}_{o}$ characterized by the second-order harmonics at $\text{2}F_{o}$ and perturbation percentage of $I_{o}$ can be approximately represented by Eq (19).


$$G_{V_{DC}\varphi \_SPS}=\frac{{\tilde{V}}_{DC}}{\tilde{\varphi }}=\frac{V_{IN}R_{inv\_eq}(\text{1}-\text{2}\varphi )}{\text{2}LF_{s}n\left(\text{1}+sR_{inv\_eq}C_{DC}\right)}$$ (16)

$$G_{V_{DC}\varphi \_TPS}=\frac{{\tilde{V}}_{DC}}{\tilde{\varphi }}=\frac{V_{IN}R_{inv_{eq}}(D_{\text{1}}+D_{\text{2}}-\text{2}\varphi )}{\text{4}LF_{s}n\left(\text{1}+sR_{inv\_eq}C_{DC}\right)}$$ (17)

$$G_{V_{DC}I_{o}}=\frac{{\tilde{V}}_{DC}}{{\tilde{I}}_{o}}=R_{inv_{eq}}$$ (18)

$${\tilde{I}}_{o}=I_{o}\left[\frac{V_{DC}}{R_{{inv}_{eq}}}\mathrm{\backslash rightleft}[\frac{\text{2}\pi (\text{2}F_{o})}{s^{\text{2}}+{\left[\text{2}\pi (\text{2}F_{o})\right]}^{\text{2}}}\right]$$ (19)

$$G_{PI}=K_{P}+\frac{K_{I}}{s}$$ (20)

**Fig 9:**
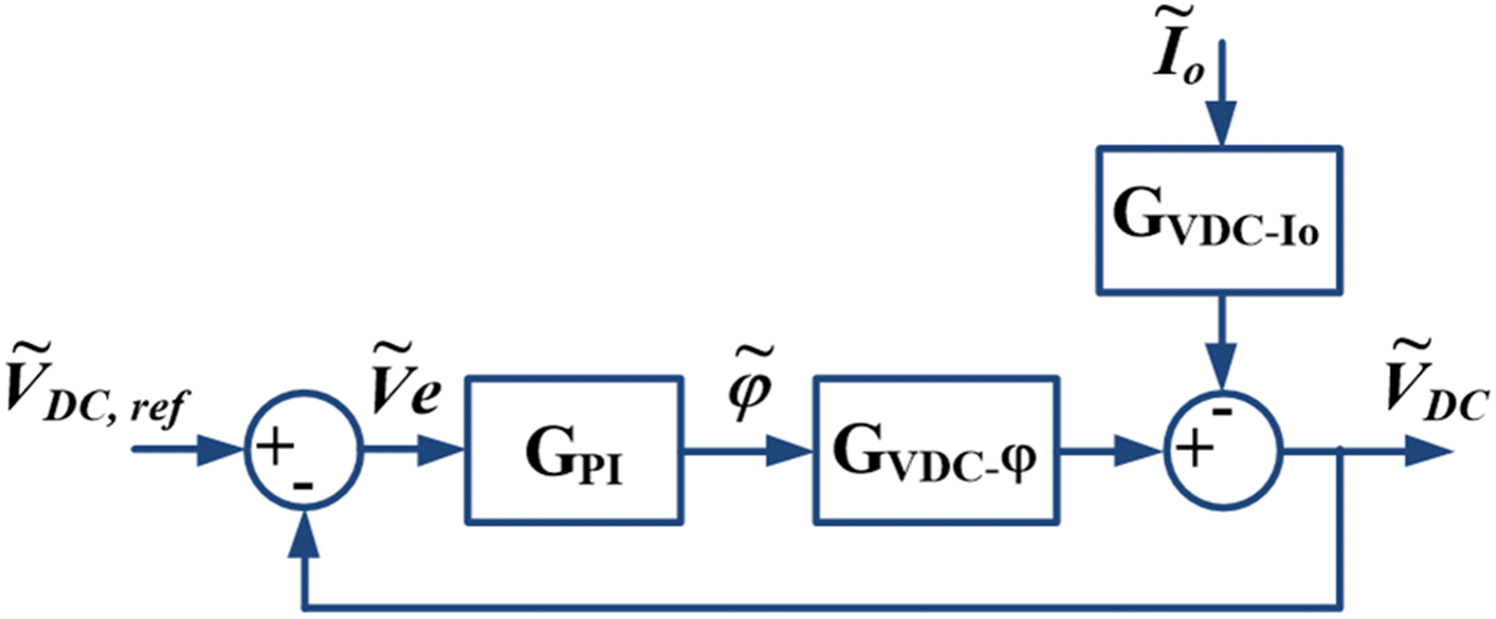
Small-signal closed-loop control of DAB with an inverter load.

Finally, the small signal closed-loop control of the DAB converter with single-phase inverter output can be obtained and shown in Fig 9. From the figure, $G_{PI}$ is the transfer function of the PI controller given by Eq (20). It is noted that the $G_{V_{DC}\varphi }$ for SPS or TPS is selected accordingly for analysis based on the operating power of the DAB converter.

## 3 Simulation results

**Fig 10:**
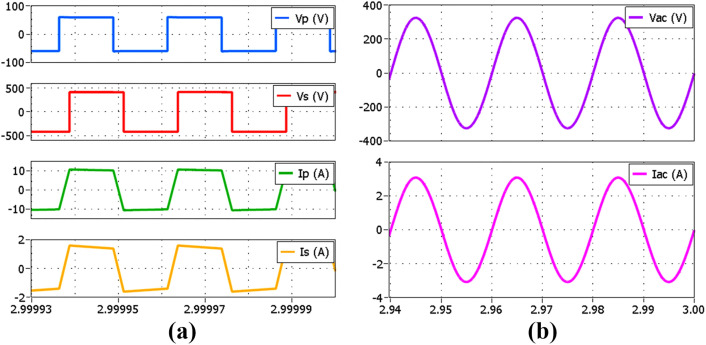
Simulated voltage and current waveforms at output power of 500 W. (a) DAB using SPS (b) Inverter.

**Fig 11:**
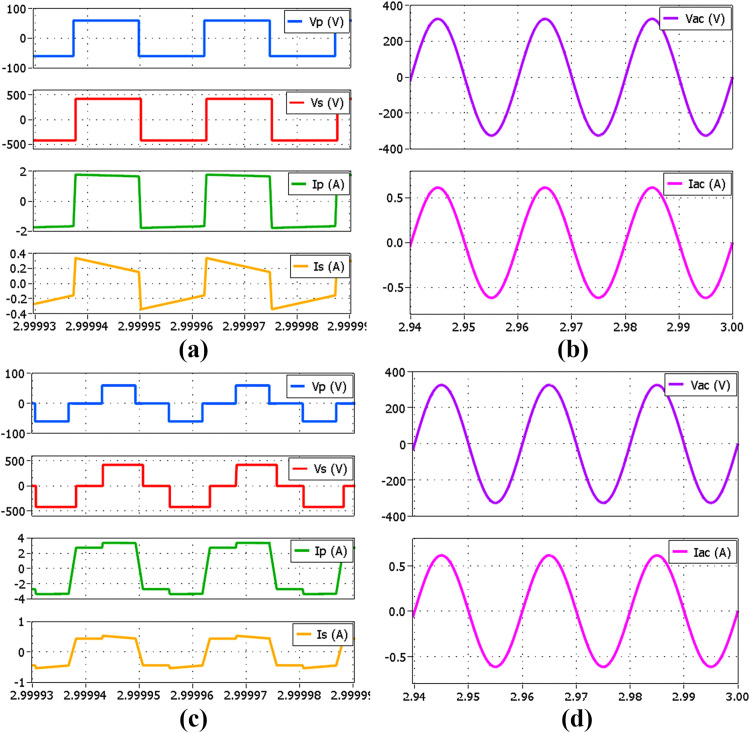
Simulated voltage and current waveforms at output power of 100 W. (a) DAB using SPS (b) Inverter connected to DAB using SPS (c) DAB using TPS (d) Inverter connected to DAB using TPS.

**Fig 12:**
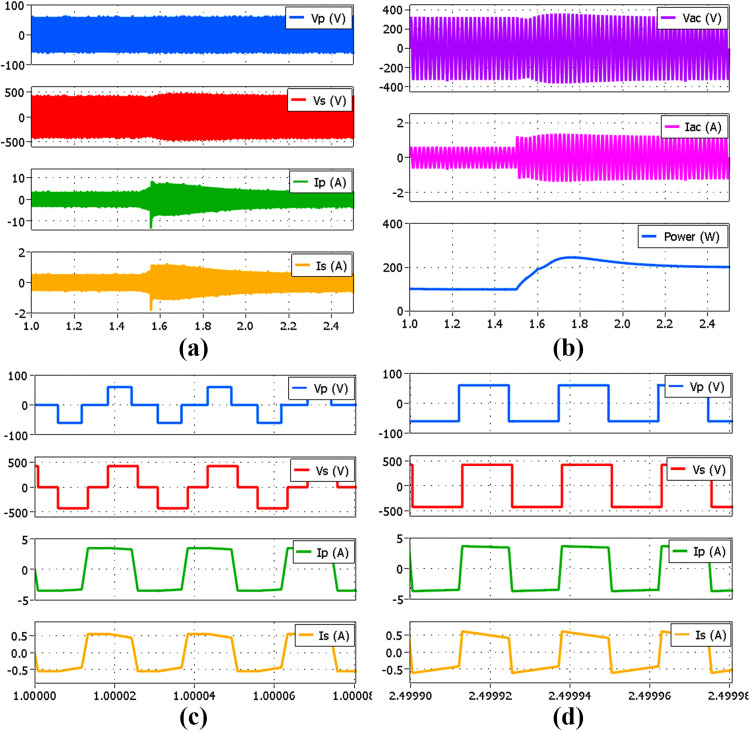
Simulated transient performance for a load step from 100 W to 200 W at t = 1.5 s. (a) DAB voltage and current (b) Inverter output voltage, current and power (c) Zoom-in view of DAB voltage and current under TPS (d) Zoom-in view of DAB voltage and current under SPS.

Further analysis of the proposed simplified hybrid modulation method is performed through simulation by using Plexim’s PLECS 4.7.5 software. The circuit configuration in Fig 1 is modeled together with the proposed controller shown in Fig 6. The analyses performed include transient and steady-state performance at several power levels comprising both SPS and TPS modulations. Fig 10 shows the voltage and current waveforms of the DAB and the inverter at the rated 500 W power output using SPS modulation. $V_{P}$ and $I_{P}$ are the primary full-bridge voltage and current, $V_{S}$ and $I_{S}$ are the secondary full-bridge voltage and current, whereas $V_{AC}$ and $I_{AC}$ are the inverter output voltage and current. Meanwhile, Figs 11(a) and 11(b) show the voltage and current waveforms of the DAB and the inverter at 100 W power output using SPS and TPS modulations respectively. Both modulation methods produce the same AC outputs despite the differences in the full bridges’ voltage and current. In the proposed hybrid modulation method, a hysteresis controller is used to switch the DAB operation between TPS and SPS when the output power exceeds the specified boundary limit, which is 30% of the rated power or 150 W. To test the transient performance when the modulation mode is changed, the load is increased from 100 W to 200 W at t=1.5 s as shown in Fig 12. It is observed that an overshoot momentarily occurs when TPS modulation changes to SPS before achieving the steady-state conditions.

**Fig 13:**
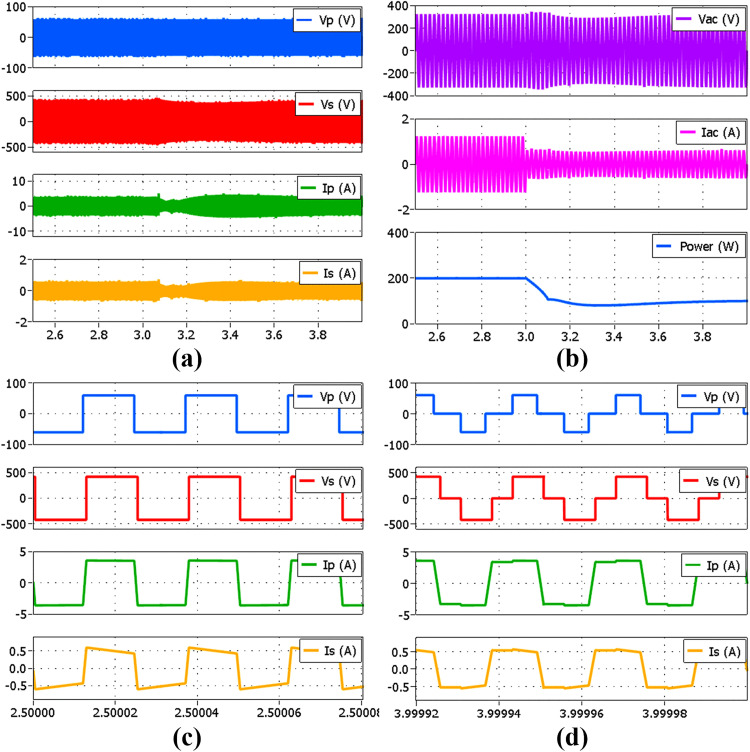
Simulated transient performance for a load step from 200 W to 100 W at t = 3 s. (a) DAB voltage and current (b) Inverter output voltage, current and power (c) Zoom-in view of DAB voltage and current under SPS (d) Zoom-in view of DAB voltage and current under TPS.

Fig 13 illustrates the transient performance in case the load is decreased from 200 W to 100 W at t=3 s. Despite the overshoot occurrence during transients, the steady state condition is satisfactorily achieved when the modulation mode has completely changed. Nevertheless, the impact is minimal as the switching only occurs at low power conditions. The same PI controller parameters are applied to both modulation modes, with $K_{P}=\text{0}.\text{8}$ and $K_{I}=\text{4}$, in which these parameters are tuned using the Ziegler-Nichols method. From the small signal model developed in Fig 9, the closed-loop stability analysis is performed based on the DAB parameters listed in Table 3 with a 5% small signal perturbation to the load current.

**Table 3:** Specifications of the DAB Converter.

Parameter	Value
DC Input Voltage, $V_{IN}$	60 V
DC Output Voltage, $V_{DC}$	420 V
Switching Frequency, $F_{S}$	40 kHz
Output Power, $P_{DC}$	500 W
Inductor, L	12 μH
Capacitor, $C_{DC}$	390 μF
Switching Device, LV	Infineon IRF150P221AKMA1
Switching Device, HV	Wolfspeed C3M0065090D
Transformer Core	Ferrite E55 N87
Transformer Ratio ($N_{P}:N_{S}$)	1:7

**Fig 14:**
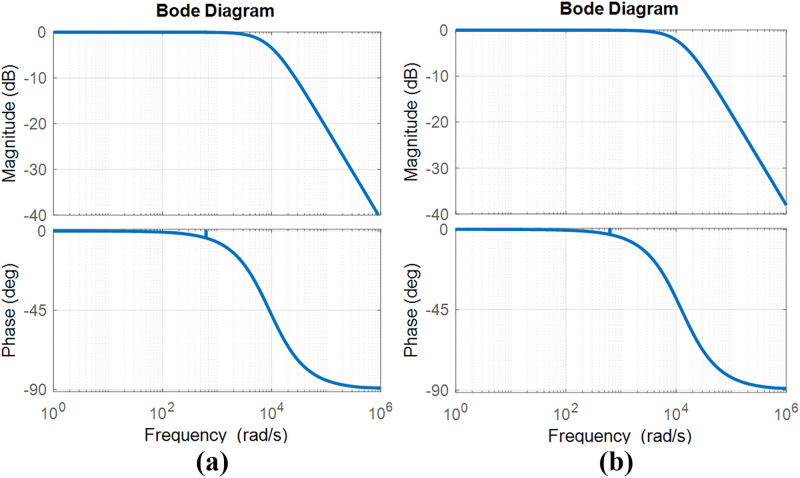
Bode plots of the closed-loop control of the small-signal model in Fig 9 at different output powers and modulations. (a) 100 W using TPS (b) 500 W using SPS.

Figs 14(a) and 14(b) show the Bode diagram for the closed-loop control at the output power of 100 W using TPS and 500 W using SPS respectively. As the power level increases, the gain crossover frequency shifts to a higher value, resulting in a reduced phase margin and decreased system stability. Nevertheless, the gain and phase margins remain substantially positive, indicating that the feedback system maintains stability from low to high power levels.

## 4 Prototype design and development

**Fig 15:**
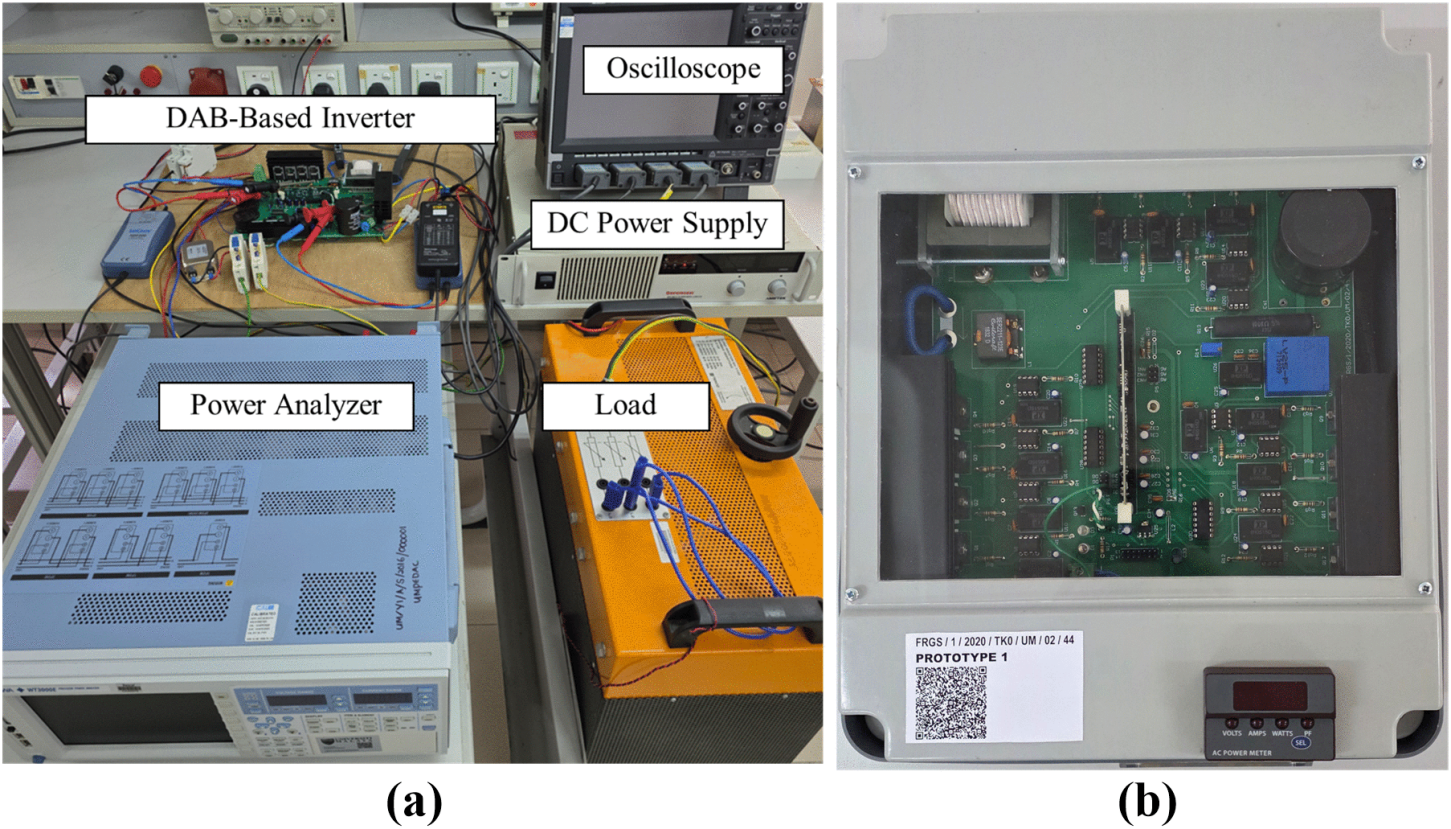
DAB-based inverter development. (a) Experimental setup (b) Completed prototype.

In order to verify the proposed simplified hybrid modulation method, a 500 W DAB-based inverter prototype has been developed. A high-speed digital signal processor (DSP) board, TMS320F28335 from Texas Instruments (TI) has been used to implement the proposed hybrid modulation for the DAB and to control the inverter. A printed circuit board (PCB) has been developed to accommodate the power circuits, TI’s DSP board, voltage and current sensors as well as the auxiliary circuits. The overall experimental setup is shown in Fig 15(a), whereas the completed prototype that shows the integrated DAB and the inverter is illustrated in Fig 15(b). The detailed specifications of the prototype are given in Tables 3 and 4.

**Table 4:** Specifications of the Inverter.

Parameter	Value
Nominal DC Input Voltage	420 V
Minimum DC Input Voltage	350 V
AC Output Voltage (RMS)	230 V
Output Frequency	50 Hz
Output Power	500 W
Switching Frequency	40 kHz
Filter inductor	4.4 *m*H
Filter capacitor	1 μF
Switching Device	Wolfspeed C3M0065090D

Potential applications of the proposed DAB-based inverter include standalone PV inverters with battery storage. With a 60 V battery pack and a required AC output of 230 V RMS, the inverter’s DC input voltage is set to 420 V based on the selected transformer ratio, n=7. This is to maintain the voltage ratio M=1 as defined in Eq (3). At rated power, the DAB operates using the SPS modulation method and therefore the required leakage inductance is estimated using the associated power equation in Eq (2). To achieve good linearity between output power and the controlling parameter, the phase shift is limited to 0.35. Considering the controller’s capability, the selected switching frequency, $F_{S}$ for implementation is 40 kHz. This means that half of the switching frequency period, represented as *T*, is 12.5 μs. Based on Eq (2), the maximum total series inductance that includes the transformer leakage inductance and external inductance required for transferring the desired output power at the maximum phase shift is 20.5 μH.

A ferrite transformer core type E55, grade N87 with an inductance factor, $A_{L}$ of 6400 *n*H and an effective core area, $A_{C}$ of 354 mm^2^ has been selected for the high-frequency transformer. With the desired input voltage and switching frequency, and by limiting the magnetizing current to 2% of the peak input current defined as twice the rated input current, the required magnetizing inductance based on Eq (21) is 723 μH. The number of turns in the primary winding, $N_{P}$ is obtained by using Eq (22) which is 10. With the transformer turns ratio of 1:7, the number of turns in the secondary winding, $N_{S}$ is 70. The magnetic flux density, $B_{m}$ within the magnetic core is estimated to be 0.11 T according to Eq (23). The measured leakage inductance of the developed high-frequency transformer is 1.3 μH. A 12 μH external inductor has been selected to enable a maximum power transfer of 500 W at a relatively low phase shift of 0.18.


$$L_{m}=\frac{V_{IN}}{\text{2}\pi F_{s}I_{m}}$$ (21)

$$N_{P}=\sqrt{\frac{L_{m}}{A_{L}}}$$ (22)

$$B_{m}=\frac{V_{\text{1}}}{\text{4}N_{P}A_{c}F_{s}}$$ (23)

The DAB output is connected to a single-phase inverter employing a unipolar sinusoidal pulse-width-modulation method. The DC input voltage applied to the inverter is regulated at 420 V by controlling the phase shifts of the DAB converter. The inverter is configured to produce an AC output voltage of 230 V RMS. The inverter output is regulated based on the relationship between the modulation index $m_{i}$, AC output and DC input, as defined in Eq (24). In this case, $m_{i}$ will be adjusted accordingly based on the actual values of the DC input voltage.


$$m_{i}=\frac{V_{AC,peak}}{V_{DC}}$$ (24)

$$L_{F}=\frac{V_{DC}}{\text{8}\Delta i_{L}F_{s}}$$ (25)

$$F_{c}=\frac{\text{1}}{\text{2}\pi \sqrt{L_{F}C_{F}}}$$ (26)

$$\text{1}\text{0}F_{o}<F_{c}<\text{0}.\text{1}F_{s}$$ (27)

To obtain a pure sinusoidal output waveform, the inverter output voltage is filtered by using an LC filter. The filter inductance, $L_{F}$ is selected based on Eq (25) with the maximum current ripple $\Delta i_{L}$ is set to 10% of the rated current. The filter capacitor is obtained by using Eq (26), where the cutoff frequency, $F_{c}$ is chosen to be 2.5 kHz according to the recommended range given in Eq (27) with $F_{o}$ is the fundamental frequency of 50 Hz.

## 5 Experimental results and discussion

The proposed simplified hybrid modulation method has been successfully tested with the developed DAB-based inverter prototype. For testing, a fixed 60 V DC was applied to the input terminal of the DAB converter to produce a 230 V RMS at the inverter output. The performance tests were conducted in both steady-state and transient conditions. Steady-state performances at various power levels were obtained by using a variable resistive load.

**Fig 16:**
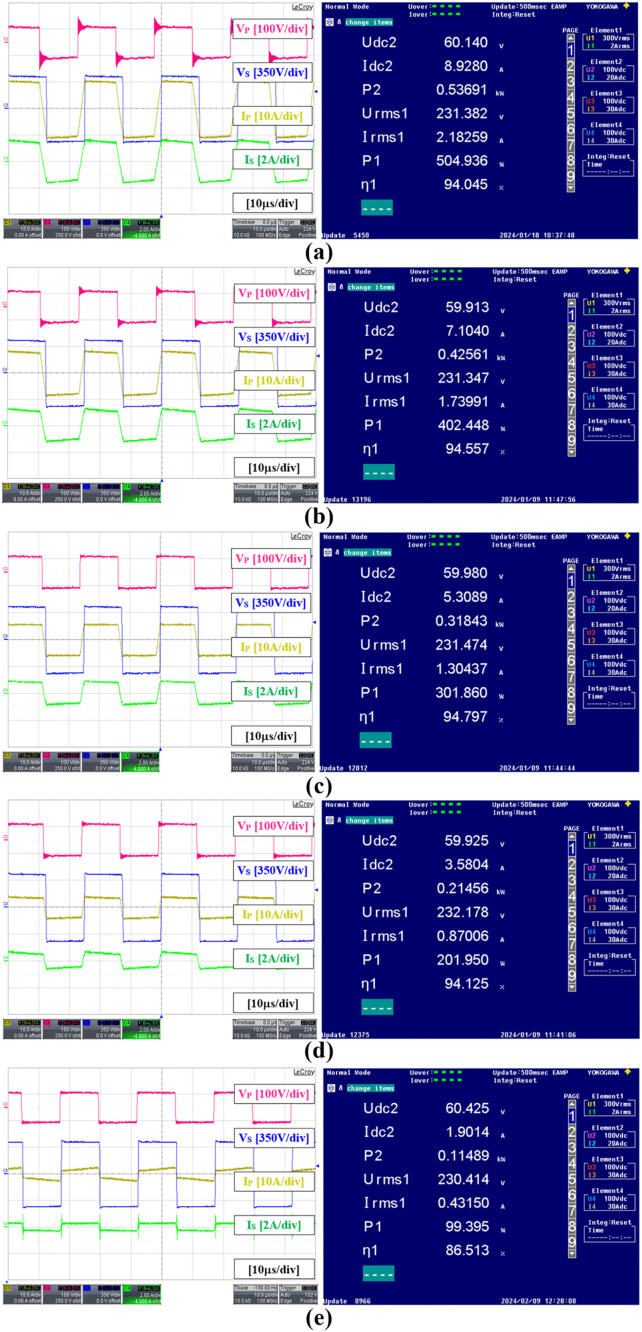
Experimental results showing voltage and current waveforms within the DAB under SPS modulation, with detailed power analyzer measurements. (a) 500 W (b) 400 W (c) 300 W (d) 200 W (e) 100 W.

Fig 16 shows the full bridges’ voltage and current waveforms within the DAB converter together with the detailed measurements obtained by using the Yokogawa WT3000 Precision Power Analyzer for an output power varying from 500 W to 100 W by using the SPS modulation method. The results were acquired instantaneously when the AC output power reached approximately 1% of the desired value. The power analyzer readings Udc2, Idc2, and P2 are the DAB input voltage, current, and power respectively measured using Channel 2. Whereas, Urms1, Irms1, and P1 are the AC output voltage, current, and power respectively measured using Channel 1. The DC to AC conversion efficiency is denoted by η1. It can be seen that, as the power level decreases the current amplitude in both primary and secondary full bridges also decreases, but maintains the same voltage amplitude for both $V_{P}$ and $V_{S}$.

**Fig 17:**
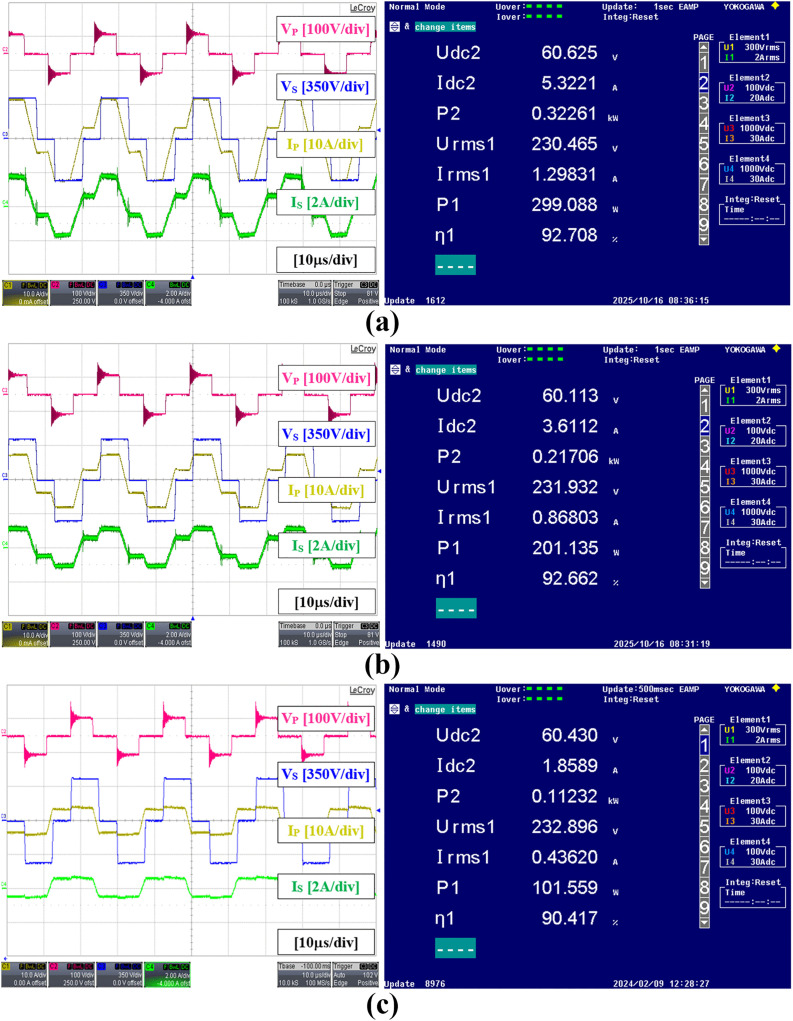
Experimental results showing voltage and current waveforms within the DAB under TPS modulation, with detailed power analyzer measurements. (a) 300 W (b) 200W (c) 100 W.

**Fig 18:**
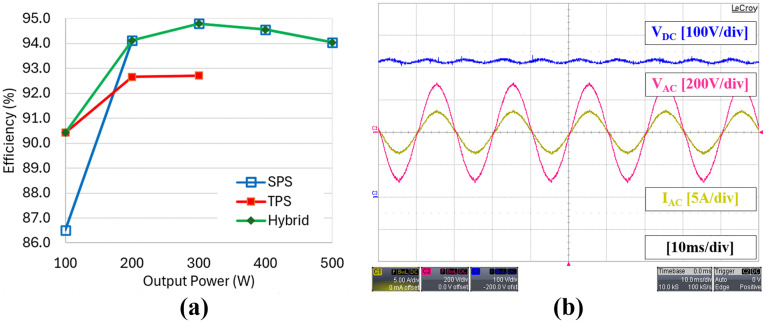
Experimental results of inverter output performance. (a) DC-AC conversion efficiency versus output power using the proposed hybrid modulation method combining TPS and SPS (b) Voltage and current waveforms at rated output power of 500 W.

Fig 17 shows the results when the TPS modulation method is used for an output power varying from 300 W to 100 W as indicated by the three-level voltage waveforms in both primary and secondary sides of the DAB converter. It is noted that the maximum output power under TPS modulation is limited to 300 W due to the restriction in the high-frequency transformer design and to prevent it from being saturated. It can be seen from Fig 17(a) that the primary current amplitude under TPS at 300W is higher than the primary current amplitude under SPS at 500W in Fig 16(a). The graph showing the DC to AC efficiency of the proposed hybrid modulation method which combines the efficiencies of both pure SPS and the selected TPS modulations is given in Fig 18(a). It is noted that the efficiency is calculated based on the AC output power from the inverter output and the DC input power at the DAB converter input. As expected, the conversion efficiency at a low power level of 100 W is higher under the TPS modulation, 90.42% compared to the SPS modulation, 86.51%. This enhancement in efficiency is attributed to ZVS operation, despite a slight increase in the amplitudes of the primary and secondary currents. Although the efficiency of TPS increases with power, it remains lower than that of SPS within the possible test range. This is because only the specific TPS mode is applied under the proposed hybrid modulation. In a pure TPS implementation as discussed in [20] and [21], the different TPS modes were employed to obtain the highest possible efficiency compared to SPS as the power increases, but at the expense of greater implementation complexity. In this paper, the highest efficiency is achieved through SPS modulation when the power level is at 300 W which is 94.80%.

**Fig 19:**
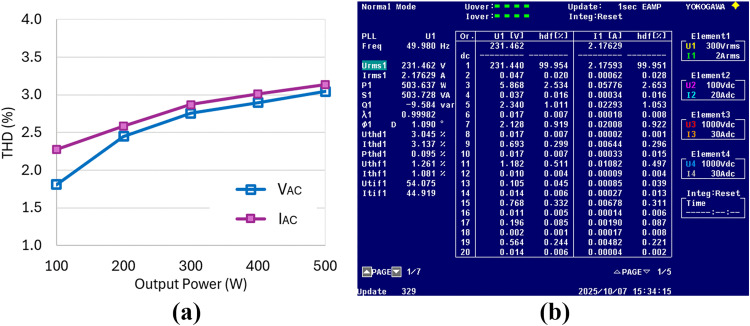
Experimental results of inverter output performance. (a) Voltage and current THD versus output power using the proposed hybrid modulation method (b) Detailed voltage and current harmonics at rated output power of 500W.

The developed DAB-based inverter prototype produces a pure sinewave output as illustrated in Fig 18(b), which shows the inverter output voltage, $V_{AC}$ and current, $I_{AC}$ at the rated output power of 500W. The THDs of both the inverter output voltage and current throughout the power range are presented in Fig 19(a), while the detailed harmonic contents at the rated power level are shown in Fig 19(b). The measured THDs for voltage and current at rated power are 3.045% and 3.137% respectively. Overall, both THD values remain below 5% under all operating conditions. However, the observed increase in harmonic content from low to high power levels is due to the rising amplitude of the second-order harmonics present in the DC link voltage, $V_{DC}$, as illustrated in Fig 18(b).

**Fig 20:**
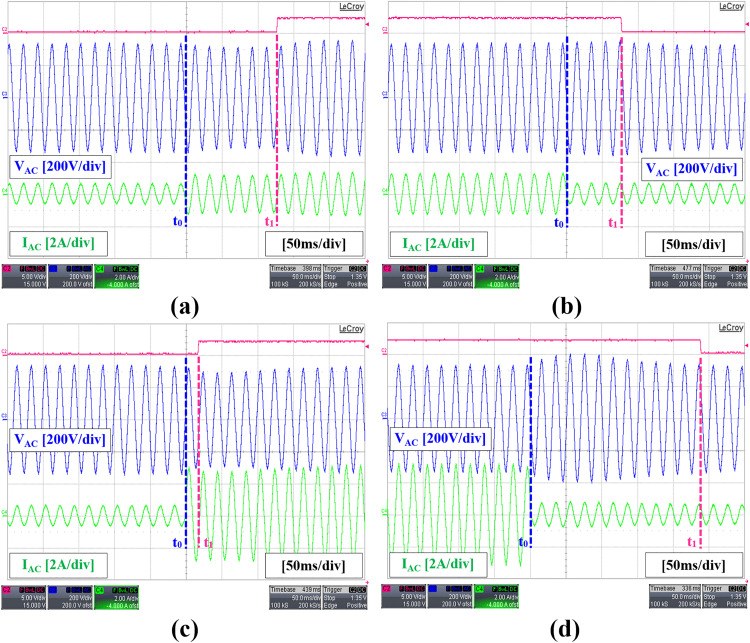
Experimental result of the inverter transient performance with a step change in load. (a) 100 W to 200 W (b) 200 W to 100 W (c) 100 W to 500 W (d) 500 W to 100 W.

In the proposed hybrid modulation method, the low power level is defined at 30% of the rated power which means that the power boundary is 150 W. Since the hysteresis controller is used to control the modulation, the upper and lower limits of the hysteresis controller are set to 150 W ± 5% to prevent frequent switching mode transitions especially when the output power is very close to the boundary. The transient performance during modulation changes was tested by applying step increases and decreases in load. Since the proposed method emphasizes efficiency as a benefit of reduced complexity, there will be a compromise in the dynamic response of the DAB converter. It is noted that modulation transitions between TPS and SPS are initiated only upon reaching the upper or lower threshold of the hysteresis controller, as indicated by high and low digital signals in Fig 20, resulting in a transient delay that constitutes a limitation of the proposed hybrid modulation method. Referring to Fig 20, the modulation change only occurs at $t=t_{\text{1}}$, a few cycles after the load change is initiated at $t=t_{o}$. The response varies depending on the amplitude of the power level change. Faster response is observed when switching from TPS to SPS at larger amplitude differences, as evidenced in Fig 20(c), in comparison with Fig 20(a). In contrast, the response is slower when switching from SPS to TPS under similar conditions, as shown in Fig 20(d), in comparison with Fig 20(b). The inverter output voltage remains well-regulated during transitions between TPS and SPS, despite minor fluctuations.

**Fig 21:**
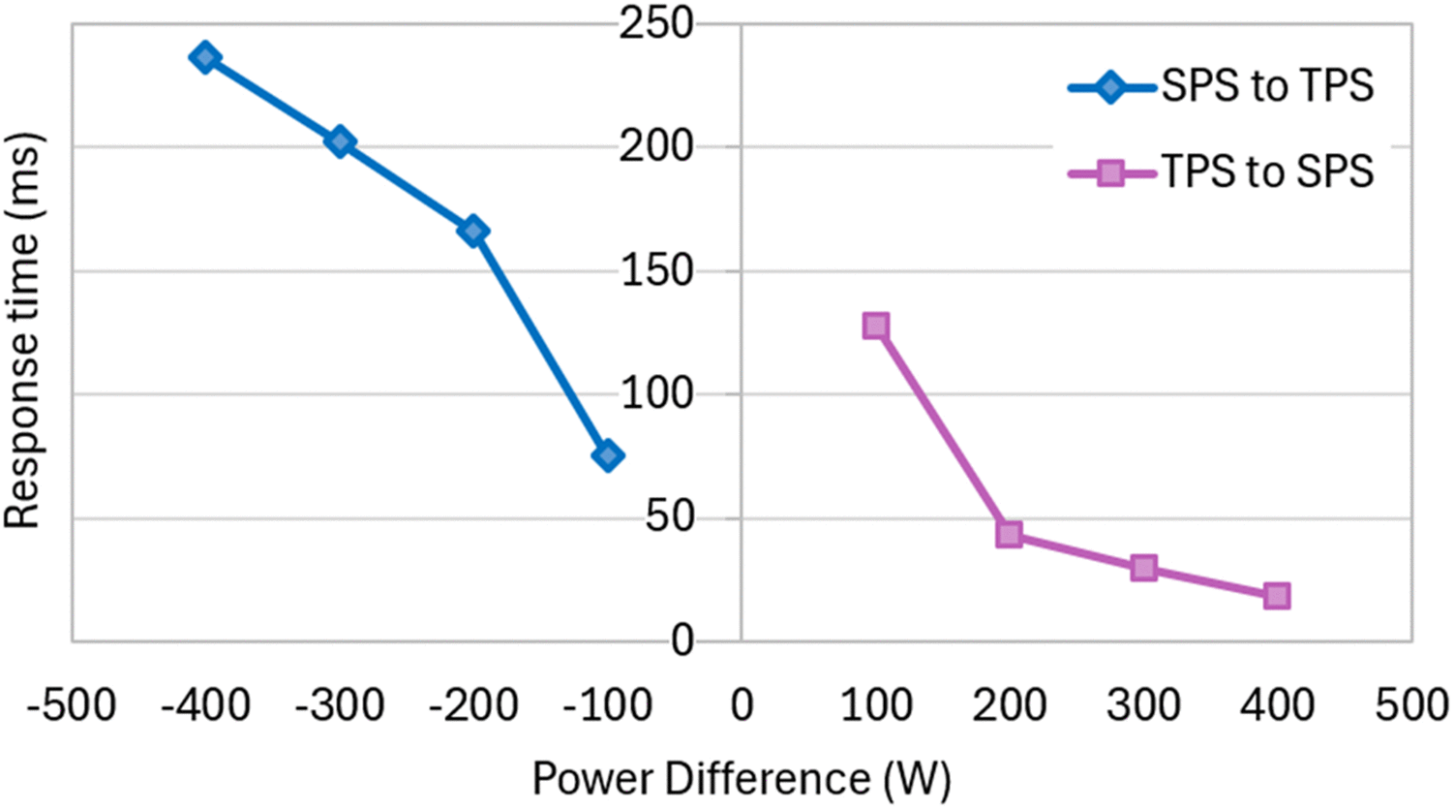
Measured response time under various step changes in output power levels.

Fig 21 presents the result of a sensitivity analysis conducted showing the response time for various step changes in output power levels from 100 W to 200 W, 300 W, 400 W, and 500 W, and vice versa. Positive and negative power differences correspond to stepwise increases and decreases in load, respectively. This finding shows that the proposed simplified hybrid modulation method maintains good control stability or robustness despite minor transient delays while offering simpler operation and good efficiency.

## 6 Conclusion

In this paper, a simplified hybrid modulation method for a DAB-based inverter has been presented. It combines the use of a specific TPS mode and SPS modulations for the DAB converter operating in low and high-power conditions respectively. The proposed modulation method uses only a single PI controller and a hysteresis controller to determine the required phase shift for output power regulation in both modulations and to regulate the TPS and SPS mode transition at the boundary power limit respectively. On the other hand, the inverter output voltage is also regulated throughout the power range regardless of the DAB converter operations. Simulation and experimental results validated the proposed modulation’s performance and functionality on a DAB-based inverter prototype under both steady-state and transient conditions. From this case study, the results indicate that high efficiency can be achieved while maintaining a simplified control system design and operation. The limitation of the proposed hybrid modulation method is the transient response delays, primarily caused by the hysteresis power limits used to determine its modulation modes. The recommended future work includes upscaling the prototypes for testing at higher power levels to evaluate the robustness of the proposed method in terms of control stability under both normal and fault conditions, including thermal management requirements. In addition, an adaptive control or gain scheduling method could be explored to improve the output power regulation with a faster dynamic response.

## Supporting information

S1 Table Efficiency, THD, and sensitivity analysis data. (DOCX)
