# Supplementary material for: Design and performance analysis of a simplified hybrid modulation method for dual active bridge powering inverters
Source: PLoS One. 2026 Feb 3;21(2):e0341443. doi: 10.1371/journal.pone.0341443 (PMC12867257; doi:10.1371/journal.pone.0341443)
Supplement: S1 Table — (DOCX) [file pone.0341443.s001.docx]

**SUPPORTING INFORMATION**

**S1****A Table. Efficiency data for Fig. 18(a).**

| **Output Power (W)** | **SPS (%)** | **TPS (%)** | **Hybrid (%)** |
| --- | --- | --- | --- |
| **100** | 86.513 | 90.417 | 90.417 |
| **200** | 94.125 | 92.662 | 94.125 |
| **300** | 94.797 | 92.708 | 94.797 |
| **400** | 94.557 |  | 94.557 |
| **500** | 94.045 |  | 94.045 |

**S1B Table.** **THD data for Fig. 19(a).**

| **Output Power (W)** | **THD_VAC_ (%)** | **THD_IAC_ (%)** |
| --- | --- | --- |
| **100** | 1.811 | 2.278 |
| **200** | 2.451 | 2.588 |
| **300** | 2.757 | 2.871 |
| **400** | 2.898 | 3.009 |
| **500** | 3.045 | 3.137 |

**S1C Table.** **Sensitivity Analysis data for Fig. 21.**

| **Power Difference (W)** | **SPS to TPS Response (ms)** | **TPS to SPS Response (ms)** |
| --- | --- | --- |
| **-400** | 236.4 |  |
| **-300** | 202.3 |  |
| **-200** | 165.9 |  |
| **-100** | 75.0 |  |
| **100** |  | 127.3 |
| **200** |  | 43.2 |
| **300** |  | 29.5 |
| **400** |  | 18.2 |
